# Utilizing Graphical Analysis of Chest Radiographs for Primary Screening of Osteoporosis

**DOI:** 10.3390/medicina58121765

**Published:** 2022-11-30

**Authors:** Soichiro Saeki, Kouichi Yamamoto, Rie Tomizawa, Szilvia Meszaros, Csaba Horvath, Luca Zoldi, Helga Szabo, Adam Domonkos Tarnoki, David Laszlo Tarnoki, Takayuki Ishida, Chika Honda

**Affiliations:** 1 Center Hospital of the National Center for Global Health and Medicine, Tokyo 162-8655, Japan; 2 Department of Global and Innovative Medicine, Graduate School of Medicine, Osaka University, Osaka 565-0871, Japan; 3 Department of Radiological Sciences, Faculty of Health Sciences, Morinomiya University of Medical Sciences, Osaka 559-8611, Japan; 4 Center for Twin Research, Graduate School of Medicine, Osaka University, Osaka 565-0871, Japan; 5 School of Nursing, Graduate School of Nursing, Osaka Metropolitan University, Osaka 545-8585, Japan; 6 Department of Internal Medicine and Oncology, Semmelweis University, 1082 Budapest, Hungary; 7 Medical Imaging Centre, Semmelweis University, 1082 Budapest, Hungary; 8 Department of Medical Physics & Engineering, Graduate School of Medicine, Osaka University, Osaka 565-0871, Japan; 9 Department of Public Health Nursing, Shiga University of Medical Science, Otsu 520-2192, Japan

**Keywords:** bone fractures, bone mineral density, prevention, aging society, Japan, Hungary

## Abstract

*Background and Objectives*: Osteoporosis is a major risk of fractures, harming patients’ quality of life. Dual-energy X-ray absorptiometry (DXA), which can detect osteoporosis early, is too expensive to be conducted on a regular basis. Therefore, we aimed to evaluate a screening method using chest radiographs developed in Japan applied to another population. *Materials and Methods*: Fifty-five patients who had a chest radiograph and DXA and applied within three months of each test were recruited from the patient database of Semmelweis University (Budapest, Hungary). Graphical analysis of the chest radiographs was conducted to identify the ratio of the cortical bone in the clavicle of each patient. Two researchers performed the analysis, and multiple regression was conducted to determine the bone mineral density of each patient provided by DXA. *Results*: The Pearson correlation between two examiners’ determinations of the cortical bone ratio was 0.769 (*p* < 0.001). The multiple regression model proved to be statistically significant in identifying osteoporosis, but the model adopted for the Hungarian population was different compared to the Japanese population. *Conclusions*: This simple, economic Japanese graphical analysis method for chest radiographs may be feasible in detecting osteoporosis. Further studies with a larger population of patients with greater variety of ethnicity would be of value in improving the accuracy of this model.

## 1. Introduction

Osteoporosis is associated with decreased bone mass, associated microarchitectural deterioration, and fragility fractures [1]. It is a widespread disease affecting mainly elderly patients, and it is associated with inadequate nutrition, inappropriate calcium and vitamin D intake, irregular menstrual cycles, and lack of physical exercise. Osteoporosis remains a significant public health problem, mainly because it is largely underdiagnosed and undertreated [2]. Osteoporosis is indicated to have a major impact on mortality [3] as well as harming patients’ quality of life [4] by making them more “immobile” [5]. In Japan, as the population ages, cases of osteoporosis have been on the rise according to a recent local study [6], and is believed to continue to increase—not only limited to the female population [7]. The prevalence of osteoporosis is estimated to be high in high-income countries [8] other than Japan as well.

The gold standard for diagnosing osteoporosis is the measurement of bone mineral density (BMD) by dual-energy X-ray absorptiometry (DXA). However, DXA requires specific, expensive equipment, as well as well-trained technicians. Furthermore, Medicare payments have cut DXA checkup for osteoporosis [9], leading to low rates of DXA screening in the United States [10]. Hence, the necessity for a new, low-cost, simple screening method of osteoporosis is of great necessity.

Previously, Kumar and Anburajan [11] reported a method of grouping patients into low and high BMD from the clavicle cortical bone length ratio. As this method required a quantitative method to determine the margins between the cortical and cancellous bone, Ishikawa et al. [12] reported a method for determining the BMD of patients from the clavicle by graphical analysis. However, this method has only been validated among patient data in Japan, which utilizes the young adult mean as the key indicator for the diagnosis of osteoporosis [13,14], unlike the other countries using Z-scores and T-scores [14]. Therefore, we conducted a study to evaluate a method of screening chest X-rays obtained from patients in Hungary to comply with global standards.

## 2. Materials and Methods

### 2.1. Subjects, Study Design

This is a single-centered, retrospective study conducted in Hungary. The study data were derived from patient records and analyzed for secondary use. From the patient database of Semmelweis University (Budapest, Hungary), 55 patients who had a chest radiograph and DXA and applied within three months of each test were identified and included in this study. Chest radiographs were performed on the following X-ray devices: 7X PRO 100-HF 650 (7x Orvostechnika Ltd., Budapest, Hungary), 7X Super 750B (7x Orvostechnika Ltd., Budapest, Hungary), GE Discovery XR 656 (GE Healthcare, Milwaukee, WI, USA).

**Figure 1:**
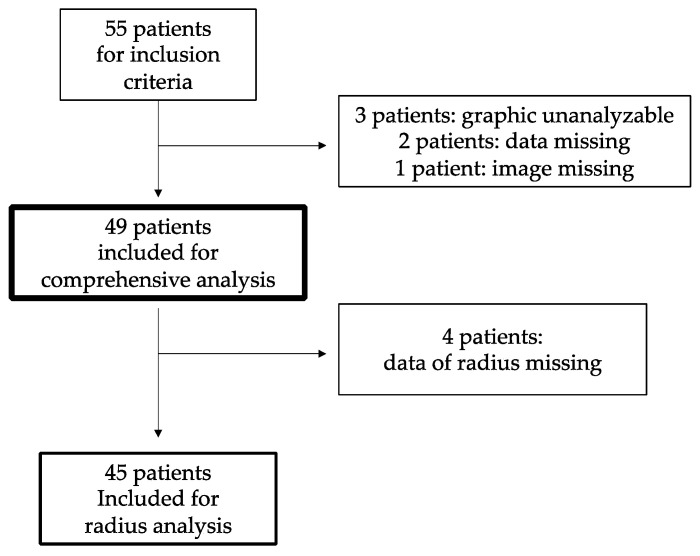
Inclusion and exclusion criteria.

Bone mineral density was measured with GE Lunar Prodigy (GE Healthcare, Milwaukee, WI, USA) dual-energy X-ray absorptiometry. As shown in Figure 1, a total of six patients were excluded: three patients as optimal location for the analysis could not be identified; two patients as fundamental data were missing; and one patient as the patient’s chest radiograph could not be identified. In addition, of the 49 patients included in the study, four patients were found with data of the left radius missing and were excluded from the analysis of the radius. 

This study protocol was approved by the ethical committee of the Graduate School of Medicine, Osaka University (approval number: 15569-6, approved 17 August 2022) and the Semmelweis University Regional and Institutional Committee of Science and Research Ethics (approval number: 14/2019, approved 15 February 2019).

### 2.2. Graphical Analysis

Graphical analysis was conducted under the methods developed by Ishikawa et al. [12] 

First, the shade of the clavicle was extracted from the chest X-ray. The contrast-limited adaptive histogram equalization conducted by using open source library Fiji v 1.51i [15] created by the National Institute of Health for ImageJ to increase the contrast and to conduct Canny edge detection.

**Figure 2:**
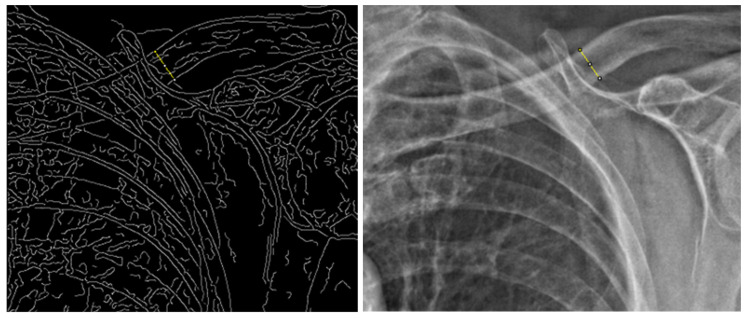
Graphical analysis conducted with Fiji. The region of interest (yellow line) was determined using Canny edge detection, while the pixels were analyzed under enhanced local contrast.

After the filtering, a region of the proximal clavicle where the clavicle became horizontal to the axis of the radiogram was determined by visual judgement, and the edge pixels were extracted by hand. The procedures for drawing perpendicular lines across the upper and lower margins of the clavicle were decided so that the line was between 90 ± 10 degrees from the lines that were drawn to the lower margin of the clavicle from its origin (Figure 2).

The pixels defined on the perpendicular line from the previous step were used to create a pixel value profile, and an eight-order function was used to fit the approximated curve. The gradients of the tangent lines for the approximated curve were defined in the areas between the upper and lower clavicle margins. The first part where the tangent gradient became the minimum after the first local maximal value was defined as the upper margin of the cortical bone and the cancellous bone, and the distance between that point from the upper clavicle margin was defined as the upper clavicle cortical bone length (CL). The point over the local minimal value with the largest tangent gradient was defined as the lower margin between the cortical bone and the cancellous bone, and the distance between the point of the lower clavicle margin was defined as the lower CL. Clavicle cortical bone-length ratio (CLR) was defined by dividing the upper CL and lower CL by the width of the short axis of the clavicle. From the pixel value profile defined from the previous step, the average CLR was adopted from the three lines used for the analysis.

### 2.3. Statistical Analysis

We presented each continuous variable with its mean and standard deviation (SD), and each categorical variable is represented by numbers and percentages. A *t*-test was applied to the continuous variables, while a chi-squared test was applied to the categorical variables.

Two independent examiners were assigned to analyze the radiographs. The two examiners conducted their analysis on each side of the clavicle of each patient. Pearson correlation analysis was conducted to validate the results of the CLR between the two examiners. A multiple regression logistics analysis was conducted using the parameters sex, CLR, age [year], body weight [kg], height [cm], BMI (body mass index) [kg/m^2^].

EZR (Saitama Medical Center, Jichi Medical University, Saitama, Japan) [16], a graphical user interface for R (version 3.6.1) [17] was used to perform statistical analysis. *p*-value less than 0.05 was determined as statistically significant.

## 3. Results

Table 1 describes the demographics of the patients included in the study. The average age was 65.3 years, and the average BMI was 26.3. The Pearson correlation between the two examiners’ measured CLR was 0.769 (*p* < 0.001).

Results of the DXA and Z-scores obtained from the DXA are shown in Table 2. The study population showed an average Z-score less than zero in all four regions of the DXA measurement.

The results of the logistic regression analysis are shown in Table 3 (L2-4), Table 4 (femoral neck), Table 5 (total femur), and Table 6 (radius). The coefficients serve as the variables that estimate the bone mineral density of each patient.

Our model achieved statistical significance in all regions of the DXA measurement.

## 4. Discussion

This study aimed to develop a method to utilize chest radiographs for the primary screening of osteoporosis. The results of our study may imply that such methods may be feasible in estimating the BMD of patients who have undergone chest X-rays, which is an important factor for the diagnosis of osteoporosis. To the best of our knowledge, no other study has been conducted to evaluate the status of osteoporosis using graphical imaging using computer analysis.

Osteoporosis is a major public health problem, affecting hundreds of millions of people worldwide. The main clinical consequence of the disease is bone fractures. It is estimated that one in three women [18] and one in five men [19] over the age of fifty worldwide will sustain an osteoporotic fracture. The majority of individuals who have sustained an osteoporosis-related fracture or who are at high risk of fracture are untreated.

Routine chest radiographs obtained for other reasons in various clinical settings can be applied to identify patients at risk of osteoporosis without additional radiation exposure or cost, which could improve osteoporosis screening. For example, in Japan, the screening rate of osteoporosis for women remains low at 4.6% [20], and this simple approach could pave way to identifying potential patients, especially where DXA is not widely available. As DXA requires expensive equipment compared to chest radiographs, this method may be able to promote global health, especially in low- and middle-income countries with a comparative lack of medical resources.

Identifying high-risk groups for osteoporosis using common chest radiographs might increase the disease recognition and prevent osteoporotic fractures. A previous twin study found that BMD is strongly heritable, especially in females in all locations, which highlighted the importance of family history as a risk factor for bone fractures [21]. Public prevention programs could highlight the importance of screening, especially in such risk groups, in preventing fragility fractures. However, population-based screening for osteoporosis is still controversial and has not been implemented [22]. The North American Menopause Society released a position statement on the management of osteoporosis in postmenopausal women in 2021 to reaffirm the importance of screening and assessing risk factors of fractures [23]. Various national societies also have recommendations determining which women should undergo DXA study based on the results of screening tests (questionnaires, fracture risk assessment calculators) [24]. A recent study recommends women be screened for osteoporosis beginning at age 65, while screening for osteoporosis in men should be considered based on the presence of risk factors [25]. The ROSE trial reported that the barriers to population-based screening for osteoporosis appear to be both psychosocial and physical, including factors such as aging, physical impairment, current smoking, and alcohol consumption [22]. Since chest radiographs are routinely used for lung cancer, tuberculosis, and annual workplace suitability screening in some countries among adults, the elderly, and even young populations, we believe that our program could help more efficient screening of those who are at risk of osteoporosis. Although the chest radiography’s graphical analysis could not replace DXA for BMD screening, it could be used where DXA has not been performed and chest radiography is readily available.

Our study has two strengths that support the feasibility of the method created by Ishikawa et al. [12]. Firstly, our analysis has been conducted on patients other than the Japanese population. The prevalence of osteoporosis differs from country to country [8], and our manuscript would add to the previous evidence of the Japanese population with another European population. Furthermore, recent evidence has demonstrated that there are health disparities among a variety of diseases [26,27,28], and this study would also concur with such research. Secondly, we were able to validate the methods with two independent examiners, while the analysis by Ishikawa et al. [12] was conducted by only one personnel member. The strong correlation between the two examiners’ results implies that this method may be feasible for different institutions. These two strengths imply that this method may be feasible for different races and ethnicities, and different examinees, which are both important potentials for this method to be applied in clinical situations.

This study has several limitations. Firstly, the nature of the study limits the participants to a relatively osteoporotic population, as the participants were recruited from a selection of hospital patients. This can be seen by the relatively low average of Z-scores (less than zero). Thus, a relatively healthy population may not have been able to participate in our study. However, as our method is thought to be used to screen relatively ill patients, we believe that this aspect of our study may be viewed as a strength, rather than a limitation. Secondly, our model derived from multiple logistic regression could not be verified on a clinical basis, as we were unable to obtain any data that recorded the Z-score of the BMD of the Hungarian population. The results obtained were automatically derived from the measurement machine, and the manufacturer was not able to provide the authors with such data. Thirdly, the sample size of the study remains relatively small, which may have been a reason for reporting models with covariates that were not statistically significant. Fourthly, this study is limited to the Hungarian population. As both genetic and environmental factors are thought to play a role in bone mineral density as well as fractures [21], future studies should include more participants, possibly from a variety of races and ethnicities, from various regions. It may be of interest to conduct studies of race and ethnicity that may not be the majority of the population to identify the influence of environmental factors associated with osteoporosis, although such planning would require substantial effort [29]. Furthermore, as this system relies on a human researcher to conduct the study; this process may be replaceable by artificial intelligence (AI) or machine learning, which could speed up the screening process.

## 5. Conclusions

In conclusion, our study revealed that a Japanese graphical analysis method using chest radiographs may be feasible in detecting osteoporosis. This method does not require the use of DXA and would be usable in areas under simple analysis. Further studies with a larger population of patients with greater variety of ethnicity would be of value to improving the accuracy of our model.

## Figures and Tables

**Table 1:** Demographics of patients included in the study.

n = 49 (Male 15)	Average	SD	Min	Max
Age	65.3	13.4	25.0	85.0
BMI	26.3	5.8	17.0	39.4
Height (cm)	161.3	10.4	144.0	188.0
Weight (kg)	68.3	14.7	40.0	105.0
CLR	0.25	0.067	0.15	0.41

Table legends: min: minimum; max: maximum; BMI: body mass index; CLR: clavicle cortical bone length ratio; SD: standard deviation.

**Table 2:** BMD measured by DXA with Z-scores.

		n	Average	SD	Min	Max
L2-4	(g/cm^2^)	49	1.04	0.24	0.50	1.60
	Z-score		−0.19	1.79	−3.70	3.60
Femoral neck	(g/cm^2^)		0.80	0.17	0.49	1.29
	Z-score		−0.37	1.23	−3.10	2.50
Total femur	(g/cm^2^)		0.84	0.21	0.43	1.44
	Z-score		−0.32	1.44	−4.10	3.20
Radius	(g/cm^2^)	45	0.76	0.16	0.49	1.08
	Z-score		−0.48	1.25	−4.30	1.90

Demographics of the BMD and its Z-scores for the patients included in the study. Table legends: BMD: bone mineral density; DXA: dual energy X-ray absorptiometry; SD: standard deviation.

**Table 3:** Logistic regression of the algorithm obtained from the Hungarian patients on the L2-L4.

Coefficients				
	Estimate	Std. Error	T Value	Pr (>|T|)
(Intercept)	3.128	2.30	1.361	0.18
Age (year)	0.00466	0.0027	1.725	0.09
BMI (kg/m^2^)	−0.06062	0.0435	−1.392	0.17
CLR	0.824	0.511	1.614	0.11
Height (cm)	−0.01963	0.00143	−1.374	0.18
Weight (kg)	0.03125	0.0172	1.815	0.07
Sex (male = 1)	0.159	0.0841	1.886	0.06

Other statistical values include residual standard error: 0.1986 at 42 degrees of freedom; multiple R-squared: 0.4045; adjusted R-squared: 0.32; f-statistic: 4.755 on 6 and 42 DF; *p*-value: 0.0008859. Table legends: BMI: body mass index; CLR: clavicle cortical bone length ratio; Std. Error: standard error.

**Table 4:** Logistic regression of the algorithm obtained from the Hungarian patients on the femoral neck.

Coefficients					
	Estimate	Std. Error	T Value	Pr (>|T|)	
(Intercept)	1.795	1.51	1.190	0.24	
Age (year)	0.000306	0.00177	0.173	0.86	
BMI (kg/m^2^)	−0.0113	0.00286	−0.397	0.69	
CLR	0.933	0.335	2.785	0.008	**
Height (cm)	−0.0101	0.00941	−1.070	0.29	
Weight (kg)	0.00930	0.0113	0.824	0.41	
Sex (male = 1)	0.126	0.0552	2.277	0.03	**

Other statistical values include residual standard error: 0.1303 at 42 degrees of freedom; multiple R-squared: 0.4715; adjusted R-squared: 0.40; f-statistic: 6.246 on 6 and 42 DF; *p*-value: 0.00009492. Table legends: BMI: body mass index; CLR: clavicle cortical bone length ratio; Std. Error: standard error; ** *p* < 0.001.

**Table 5:** Logistic regression of the algorithm obtained from the Hungarian patients on the femoral total Femur.

Coefficients					
	Estimate	Std. Error	T Value	Pr (>|T|)	
(Intercept)	1.416	1.93	0.734	0.47	
Age (year)	0.00112	0.00168	−2.794	0.00803	**
BMI (kg/m^2^)	−0.00679	0.0366	−0.186	0.85	
CLR	0.905	0.429	2.109	0.04	*
Height (cm)	−0.00882	0.0120	−0.733	0.4676	
Weight (kg)	0.01000	0.0144	0.692	0.4925	
Sex (male = 1)	0.153	0.0706	2.170	0.0357	*

Other statistical values include residual standard error: 0.1668 at 42 degrees of freedom; multiple R-squared: 0.4575; adjusted R-squared: 0.38; f-statistic: 5.904 on 6 and 42 DF; *p*-value: 0.0001558. Table legends: BMI: body mass index; CLR: clavicle cortical bone length ratio; Std. Error: standard error; * *p* < 0.05; ** *p* < 0.001.

**Table 6:** Logistic regression of the algorithm obtained from the Hungarian patients on the radius.

Coefficients				
	Estimate	Std. Error	T Value	Pr (>|T|)
(Intercept)	3.128	2.30	1.361	0.18
Age (year)	0.00466	0.0027	1.725	0.09
BMI (kg/m^2^)	−0.06062	0.0435	−1.392	0.17
CLR	0.824	0.511	1.614	0.11
Height (cm)	−0.01963	0.00143	−1.374	0.18
Weight (kg)	0.03125	0.0172	1.815	0.07
Sex (male = 1)	0.159	0.0841	1.886	0.06

Other statistical values include residual standard error: 0.1093 on 38 degrees of freedom; multiple R-squared: 0.6099; adjusted R-squared: 0.38; f-statistic: 9.902 on 6 and 38 DF; *p*-value: 0.0000001421. Table legends: BMI: body mass index; CLR: clavicle cortical bone length ratio; Std. Error: standard error.

## Data Availability

Due to the confidentiality of the patients’ personal information, data for this study cannot be shared.
